# Rupture of the Tendo Achillis

**Published:** 1902-01-25

**Authors:** 


### RUPTURE OF THE TENDO ACHILLIS.

Mr. Lynn Thomas, C.B., F.R.C.S., urges the advantages of treating ruptured tendo achillis by early massage and by walking about with the foot held by a metal splint so arranged along the anterior aspect of the joint as to prevent its being bent so far as to stretch the reparative material but without extension of the foot. In doing physical drill on board ship on his way out to South Africa he had the misfortune to rupture his tendon, and he treated it in the way mentioned. Having had an aluminium spatula moulded to the shape of the bend of the ankle on its anterior aspect, this was pushed into a thick indiarubber tube, which not only acted as a padding but kept the splint in its place by preventing the splint from moving downwards under the boot-lace. On the boot being put on, this anterior splint was applied, extending from the middle of the dorsum of the foot to several inches above the ankle. On lacing up the boot the lace was made to pass round and round the splint in its lower few turns. In the upper part it passed entirely in front of it. The foot was thus held firmly at a right angle. Mr. Thomas thinks that by this line of treatment a stronger union is obtained than by either immediate suturing or the fixation of the limb by splints, because the call to provide sufficient reparative callus to bind the ruptured ends of the tendons is much greater during movement in the dependent position than it is during rest in an elevated or in a fixed position. The great difference in the quantity of reparative material so produced is well shown by a comparison of human and veterinary surgery in cases of broken bones and tendons. Mr. Lynn Thomas also points out a new sign of rupture of the tendo achillis. He says that much may be learned from the shape of the surface marking produced after a few days by the extravasated blood in the various injuries which occur in the neighbourhood of the ankle, and that when the tendo achillis is ruptured there is a bilateral discolouration on each side of the tendon running downwards below both malleoli and joined at the level of the malleoli, or the seat of the ruptured tendon, by a transverse band, thus giving to the bruise a characteristic H-shape which is pathognomonic of a breach in the tendo achillis.

